# Lyme Neuroborreliosis: Mechanisms of *B. burgdorferi* Infection of the Nervous System

**DOI:** 10.3390/brainsci11060789

**Published:** 2021-06-15

**Authors:** Lenzie Ford, Danielle M. Tufts

**Affiliations:** 1 Neuroscience Research Institute, University of California, Santa Barbara, CA 93106, USA; 2 Infectious Diseases and Microbiology Department, Graduate School of Public Health, University of Pittsburgh, Pittsburgh, PA 15261, USA

**Keywords:** Lyme disease, *Borrelia burgdorferi*, infectious disease, tick-borne pathogen, post-treatment Lyme disease syndrome, neurotropism

## Abstract

Lyme borreliosis is the most prevalent tick-borne disease in the United States, infecting ~476,000 people annually. *Borrelia* spp. spirochetal bacteria are the causative agents of Lyme disease in humans and are transmitted by *Ixodes* spp ticks. Clinical manifestations vary depending on which *Borrelia* genospecies infects the patient and may be a consequence of distinct organotropism between species. In the US, *B. burgdorferi* sensu stricto is the most commonly reported genospecies and infection can manifest as mild to severe symptoms. Different genotypes of *B. burgdorferi* sensu stricto may be responsible for causing varying degrees of clinical manifestations. While the majority of Lyme borreliae-infected patients fully recover with antibiotic treatment, approximately 15% of infected individuals experience long-term neurological and psychological symptoms that are unresponsive to antibiotics. Currently, long-term antibiotic treatment remains the only FDA-approved option for those suffering from these chronic effects. Here, we discuss the current knowledge pertaining to *B. burgdorferi* sensu stricto infection in the central nervous system (CNS), termed Lyme neuroborreliosis (LNB), within North America and specifically the United States. We explore the molecular mechanisms of spirochete entry into the brain and the role *B. burgdorferi* sensu stricto genotypes play in CNS infectivity. Understanding infectivity can provide therapeutic targets for LNB treatment and offer public health understanding of the *B. burgdorferi* sensu stricto genotypes that cause long-lasting symptoms.

## 1. Introduction

Lyme disease is the most common tick-borne disease in North America and Europe, affecting over half a million people annually [1,2,3,4]. Lyme disease is caused by *Borrelia* spp. spirochetal bacteria and transmitted by *Ixodes* spp. ticks [5,6]. The most common symptoms include the presence of a skin rash (erythema migrans) at the tick bite site, as well as fever, headache, and fatigue [7,8,9,10]. If not treated with antibiotics, Lyme disease can persist and the patient may develop neurological, cardiac, chronic skin, or articular symptoms [10,11,12,13,14]. This review focuses on the later-stage neurological symptoms that can arise from Lyme disease infection in humans from North America.

The first discovery of a neurological symptom from Lyme disease infection was a 1922 finding of meningoradiculitis occurring after a tick bite [11,15]. Lyme neuroborreliosis (LNB), or Lyme disease-causing symptoms of the central and peripheral nervous system, is multifaceted and patients generally present with meningitis, cranial neuritis, radiculoneuritis, parenchymal inflammation of the brain or spinal cord, peripheral neuropathy, and/or encephalopathy [12,16,17,18]. In Europe, where the first meningoradiculitis findings occurred, Bannwarth Syndrome (also known as Garin–Bujadoux–Bannwarth syndrome) has been characterized in which LNB manifests as severe radicular pain accompanied by cranial nerve paresis [17]. Occasionally confusion and central nervous system dysfunction occur, while meningeal and encephalitic features are rare [16,19,20]. LNB is caused by the *Borrelia burgdorferi* sensu lato complex which includes the species: *B. garinii*, *B. afzelii* (common in Europe), and *B. burgdorferi* sensu stricto (ss) (common in North America) [21,22,23]. Minimal Bannwarth syndrome cases have been reported in North America and most LNB manifestations differ between continents, possibly as a result of the different genospecies of *Borrelia* that are present in these locations [8,24]. For this reason, we focus on rare North American *B. burgdorferi* ss LNB which affects 10–20% of patients [19,25,26]. The estimated number of cases in the US was nearly 2 million cases in 2020 [27]. Recently, various LNB cases have been reported in Minnesota and Wisconsin [28]; indicating that cases may be increasing in the US. In western Pennsylvania from 2003 to 2013, neurological symptoms were observed in 12% of Lyme-infected patients [29]. This study also showed that cases of pediatric Lyme disease doubled every 1.6 years [29]. Collectively, these data indicate a rising precedence of LNB in the United States and underscore the importance of studying this disease.

North American LNB largely manifests as lymphocytic meningitis, cranial neuritis, radiculoneuritis, and/or mononeuritis multiplex [9,10,11,12,13,14,16,17,19,20,22,26,30,31,32,33,34,35,36,37,38,39]. Antibodies in response to *B. burgdorferi* ss infection can persist in the cerebrospinal fluid (CSF) for several weeks or in serum for several years [40,41]. Within the first few weeks of infection, disruptions to cortex function have been measured as magnetic resonance imaging (MRI)-identified hyperintensities and positron emission tomography (PET)-identified mild-moderate hypometabolism [37,42,43]. Psychological symptoms are also present in this early stage (3–32 days), consisting of depression, decreased concentration, sleep disturbance, and memory impairment [44]. Post-*B. burgdorferi* ss infection stages (several weeks to months) can manifest as more severe neurological and psychological disruptions, including distal paresthesias, chronic encephalomyelitis, panic attacks, severe dementias, personality changes, catatonia, and mania [37,39,44]. Currently, antibiotic treatment is the only FDA-approved treatment method available for Lyme disease; however, LNB presents in the late stage of infection and tends to be resistant to antibiotics [45,46]. LNB that is antibiotic unresponsive, also called post-treatment Lyme disease syndrome (PTLDS), affects 10–20% of disease patients [17,30,39,45,46,47,48,49,50,51]. The prevalence of late-stage LNB and the lack of agreement amongst clinicians for treatment options punctuates the need for further mechanistic and translational studies of LNB.

## 2. *B. burgdorferi* ss Transmission Cycle and the Importance of Outer-Surface Proteins

The transmission of *B. burgdorferi* ss bacteria from host–tick–host is an intricate and complex process which requires spatial and temporal gene regulation of the bacteria to adapt to the vector biological environment and evasion of the host innate immune system [52,53]. White-footed mice, *Peromyscus leucopus*, are the most effective reservoir hosts of *B. burgdorferi* ss in North America; however, other small- and medium- sized mammals and several avian species may also serve as competent hosts [54,55,56,57,58,59,60,61,62,63,64,65]. Reservoir hosts generally experience persistent asymptomatic infections and *B. burgdorferi* ss may utilize various mechanisms to effectively evade the host innate immune response [65,66]. Transmission occurs predominantly via hard-bodied ticks in the *Ixodes* genus [67,68,69]. When a larval tick takes a bloodmeal from an infected host, it may acquire *B. burgdorferi* ss spirochetes. The engorged larva will molt into the nymphal life stage and will subsequently infect susceptible hosts including humans [6,10,12]. Transmission of spirochetal bacteria from tick vector to a susceptible host begins during bloodmeal uptake. Various borrelial outer surface proteins (Osp) play important roles in pathogen virulence, transmission, and survival within the host and vector. In unfed ticks, OspA and OspB are expressed and promote adherence and survival of spirochetes in the tick midgut between bloodmeals [10,70,71]. Spirochetes are sequestered in the tick midgut by expressing OspA and binding to a tick protein (TROSPA) present in tick gut epithelial cells [72,73,74]. During a bloodmeal uptake, spirochetes in the midgut begin to multiply rapidly, expression of OspA on the surface decreases, and an increase in OspC expression promotes the migration of spirochetes to the tick salivary glands [75,76,77]. OspC is crucial in the early phase of *B. burgdorferi* ss infection to evade the host innate immune system; expression decreases after 2–3 weeks in response to host antibodies [52,78,79,80,81,82]. OspC also plays a role in the attachment of spirochetes to the tick salivary protein Salp15, enhancing the spirochete load and protecting them from the host complement system by impairing the function of macrophages, neutrophils, and dendritic cells [77,83,84,85,86,87,88].

The *ospC* gene is hypervariable with genotypes varying in their genetic sequences, geographic distribution, host preferences, pathogenicity, and disease phenotypes [59,89,90,91,92]. Over 35 unique *B. burgdorferi* ss OspC genotypes are currently recognized, some of which cause the typical erythema migrans rash and others disseminate through the bloodstream or CNS and are associated with severe disease (A, B, I, K human invasive genotypes) [23,24,89,90,93,94,95,96,97,98,99], suggesting that only certain strains may exhibit neurotropism. Disease severity is dependent on several factors including genotype virulence, the abundance of spirochetes present in the tissue, co-infection with other *B. burgdorferi* ss genotypes and tick-borne pathogens, and intrinsic differences in host immune responses [21,93,97,100,101,102,103,104]. Ticks may be simultaneously infected with five or more *B. burgdorferi* ss genotypes [105,106,107,108,109]; however, the number of genotypes a tick is able to transmit during feeding is currently unclear. Certain *B. burgdorferi* ss genotypes may disseminate through the host system more rapidly than others, they may compete against each other effectively eliminating less efficient genotypes from the host, or they may facilitate infection allowing additional genotypes to thrive [110,111]. Co-infections with other tick-borne pathogens are also common in *I. scapularis* ticks and reservoir hosts (i.e., *Anaplasma phagocytophilum*, *Babesia microti*, *Bartonella* spp., other *Borrelia* spp., *Ehrlichia muris*, Powassan virus, etc.) which may also affect pathogen prevalence, persistence, and transmission efficiency [112,113,114,115,116]. *Borrelia burgdorferi* ss genotype genetic diversity is thought to be maintained through vertebrate hosts which may act as species specific niches [59,65,90,117].

## 3. Gene Specificity Allows for *B. burgdorferi* ss Infectivity of Brain Cells

*Borrelia* spp. genomes are complex, consisting of circular and linear plasmids along with a linear chromosome of ~900 kb [118]. Different species and genotypes contain a variable number of plasmids due to frequent reorganization and deletion; most of the genes required for metabolism and regulation are found on the linear chromosome, with a few protein encoding genes for growth and specific virulence factors being located on the plasmids [119,120,121,122,123]. The presence or absence of specific plasmids has been linked to infectivity in hosts [124] and specific plasmids or plasmid combinations may be necessary for LNB infection.

Various studies have identified numerous genes and proteins important for *B. burgdorferi* ss infection and host complement evasion in mammals [65,125,126,127,128,129,130]. In large part, these studies focus on skin, blood, and joint tissues. These data have provided a wealth of information that suggests strain specificity is critical to infection of different host species. *Borrelia burgdorferi* ss strains may be classified in several ways including the polymorphic *ospC* gene, the ribosomal DNA spacer restriction fragment–length polymorphism genotypes (RSTs), and the *rrs–rrlA* rDNA intergenic spacer (IGS) region [24,106,107] (Table 1). Complete classification for each *B. burgdorferi* ss strain is lacking and it is unclear how different combinations of these genes and regions may contribute to the variation in human invasive genotypes and LNB.

Spirochetal infection of the nervous system is further complicated because *B. burgdorferi* gene expression can be distinct in host blood compared to CSF [96,131,132]. For example, OspA is known to be downregulated in the skin and blood of the host, yet CSF OspA levels are upregulated in early stages of the disease [131]. In a study by Schutzer and colleagues [131], antibody levels against *B. burgdorferi* OspB and OspC increased during early infection. However, as the disease progressed to the late stage, only 23% of patients contained antibodies against OspC in their CSF. This work underscores the need for LNB studies to be measured in nervous system tissues.

## 4. Mechanisms of *B. burgdorferi* ss Entrance into the Nervous System

*Borrelia burgdorferi* ss strain specificity plays an important role in infectivity of the nervous system. While strain specificity remains a critical question in the field, various studies have identified methods of *B. burgdorferi* ss entry into the nervous system [133,134,135]. First, spirochetes enter the CNS via the bloodstream or peripheral nervous system and can be recovered from human CSF 14–18 days post-tick bite [136,137,138,139]. The presence of spirochetes in the CSF is a key factor for LNB development, as it provides an access point to the brain. Next, *B. burgdorferi* ss infiltrate the protective membranous meninges in animal models of Lyme disease [134,135], identifying the first point of brain penetration. Specifically, spirochetes are present in vascular, perivascular, and extravascular regions of the dura mater [134]. Spirochete presence is associated with an increase in T cells and leukocytes within the meninges [134,135].

The host immune response plays an important role in early stages of LNB [52,140,141,142,143,144]. Short-term infection of Rhesus macaque frontal cortex tissue by *B. burgdorferi* ss (24 h) and subsequent transcriptomic analysis identified over 2200 genes that were significantly altered, primarily those involved in immune and inflammatory response pathways [145]. Some early inflammatory responses arise from glial cells in the brain and are measured as an increase in cytokine and chemokine markers [146,147]. Alongside this early inflammation, an increased production of the pro-inflammatory cytokine astrocytic interleukin 6 (IL-6) can be measured, as well as oligodendrocyte and neuronal death [146,147,148]. *Borrelia burgdorferi* ss caused direct inflammation and apoptosis of the oligodendrocytes, but neuronal death was dependent on microglial activation [148]. These experiments identify the immediate inflammatory response in the animal brain as a result of *B. burgdorferi* ss infection, and attempt to explain the cell-type specificity that causes this inflammation.

While acute LNB is marked by the host immune response, long-term LNB may disrupt additional molecular pathways in the nervous system. Bouquet et al. [129] tracked the transcriptomic CSF profile of North American Lyme disease patients at the point of diagnosis and again 6 months after antibiotic treatment. Pre-treated transcriptomes exhibited a change in over 1000 genes, with approximately 60% upregulated. Post-treatment transcriptomes still exhibited a change in nearly 700 genes, with approximately 50% of them being upregulated. Interestingly, inflammatory response and immune cell trafficking pathways were decreased post-treatment [129]. A decrease in the inflammatory and immune pathways in the dura mater was also observed over time in a mouse model of Lyme disease [135]. This post-treatment, antibiotic-resistant stage of LNB seems to be triggered by neither inflammatory nor immune response pathways. It is this stage of LNB that is considerably understudied and requires new mechanistic insight to identify avenues for clinical intervention. Currently, the FDA-approved treatment for LNB in humans is long-term antibiotic treatment; however, numerous clinical studies have found that some patients do not recover from LNB symptoms with this treatment method [45,46]. Approximately 10–20% of patients claim to suffer from persistent Lyme disease-like symptoms months to years following appropriate antibiotic treatment. Chronic Lyme, or the more acceptable term post-treatment Lyme disease syndrome (PTLDS), is steeped in controversy as no evidence of systemic *B. burgdorferi* infection can be found in these patients and prolonged antibiotic treatments may be detrimental to the health of these patients. While a plethora of data exist on the controversies surrounding these types of patients [149,150,151,152], this is not the focus of this review.

## 5. Informative Post-Treatment Lyme Disease Genes and Antibiotic-Resistant LNB

Many studies of LNB tend to focus on the initial inflammatory symptoms of the disease, but for PTLDS, other pathways appear to be involved in the persistent neurological symptoms. Transcriptomic analysis of human CSF from 6 month post-treatment North American LNB identified that the eukaryotic initiation factor 2 (eIF2) pathway remains disrupted [129], suggesting that there may be long-term deficits in the cellular stress response of infected cells. Disruption of this pathway has been linked to a rare genetic colorectal cancer [153]. Additionally, four genes distinguished post-treatment patients with resolved symptoms from patients with persistent symptoms: *MIAT*, *CCDC163P*, *ZNF266*, and *GPR15* [129]. Of the genes identified, only *GPR15* is associated with an immune response [154]. GPR15 is a G-protein coupled receptor that acts as a chemokine receptor for human immunodeficiency virus (HIV) 1 and 2, and has been implicated in various lymphomas [154]. *CCDC163P* and *ZNF266* are involved in protein binding, with the latter possibly involved in transcription regulation [129]. *ZNF266* is also involved in nucleic acid binding, as is *MIAT* and eIF2 which bind lncRNA and tRNA, respectively [155]. *MIAT* is involved in multiple human diseases, including myocardial infarction susceptibility, schizophrenia, and substance abuse [155]. These disrupted genes are worth further investigation and may provide insight into how post-treatment Lyme disease symptoms persist in the human brain.

While extremely informative, mRNA transcriptome profiling does not provide a complete picture of LNB. There is a high likelihood that PTLDS may be regulated in part by non-coding RNAs [129]. Future studies need to expand the scope of analysis to include non-coding nucleic acids and proteins. In a cultured human astrocyte study, Casselli et al. [156] measured disruption of mRNA and miRNA levels in acute and long-term *B. burgdorferi* ss infection. Similar to Ding et al. [145] and Bouquet et al. [129], Casselli and colleagues found upregulation of mRNA involved in inflammation pathways in acute infection. The miRNA alterations, however, provided new insights; long-term infection upregulated miRNA involved in various signaling pathways and cell adhesion. The authors identified hsa-miR-143-3p as a key differentially expressed factor, as it was also involved in chronic fatigue syndrome/myalgic encephalomyelitis, which share symptoms with LNB [156]. This study highlights the importance of investigating non-coding RNAs in Lyme disease, and the long-lasting changes in RNA profiles of posttreatment LNB.

Cell culture studies raise an important consideration for post-treatment LNB experiments: are the persisting symptoms a reaction to meningeal infection or does long-term LNB arise from further spirochete penetration into the brain? In vitro, *B. burgdorferi* ss binds to neurons, glia, and extracellular matrix proteins, highlighting the possibility of penetrance beyond the blood–brain barrier [138,157,158,159,160,161]. Although spirochete presence in brain parenchyma was not observed in mouse models [135], various neurological studies of human brain find *B. burgdorferi* ss spirochete deposition in the gray matter of the cerebral cortex [162,163,164,165,166,167,168,169,170], with similar findings to those from in vitro cell culture studies [166]. These data suggest that persistent LNB symptoms may arise from alterations beyond the downstream meningeal inflammatory response and are possibly influenced by spirochete presence and *B. burgdorferi* ss DNA deposition in brain cells [159,162,163,164,168,169].

## 6. Conclusions and Future Directions

LNB is an extremely complicated disease: from the strain infectivity of the various genotypes of the *B. burgdorferi* ss spirochete, to its ability to move from the host bloodstream to the nervous system, to neurotropisms that allow penetrance of the blood–brain barrier and downstream acute inflammatory and immune response, and finally to long-lasting changes in cell adhesion and signaling pathways that coincide with spirochete deposition in the brain. Here, we propose a basic science approach to investigating post-treatment LNB.

First, there is a lack of information regarding which *B. burgdorferi* ss strains are most infectious to the human brain. The OspC genotypes A, B, I, and K are the most infectious in humans [89], but is there an OspC genotype that promotes brain infectivity? Do different combinations of *B. burgdorferi* ss genes and plasmids contribute to strain invasiveness and neurotropism? Laboratory studies often utilize the Bb-297 strain which was isolated from an LNB patient [135,171] and may not fully encompass all infectious genetic elements due to loss and reorganization of plasmids and genes over time. Furthermore, OspC is not the only Osp protein to be upregulated in human CSF after infection; OspA and OspB are also upregulated [70,71,76,131]. Additionally, *erp* genes (OspE-F-related lipoproteins) function as receptors for complement inhibitory factor H molecules and may contribute to the ability of *B. burgdorferi* spirochetes to evade the host innate immune system [172,173,174]. Investigating the role of other Osp proteins is necessary for fully understanding LNB infection, particularly OspA which can tether spirochetes to the meninges [134]. Additionally, RSTs and IGS sequences are used to distinguish strain infectivity [24,106,107], and so genotypic variations in non-coding regions must also be considered. Multilocus sequence typing and deep amplicon sequencing will be critical for better understanding the pathogenicity of certain strains and their ability to invade the human brain. Since spirochetes can deposit in the human brain and *B. burgdorferi* ss DNA can be identified in brain cells, we propose that next-generation sequencing is crucial to genotype the infectious spirochetes from human CSF and post-mortem LNB brain samples.

Second, the question remains whether post-treatment LNB results from a downstream host immune response or whether additional pathways are disrupted from spirochete penetrance into the brain. We believe that an in-depth study of acute vs. posttreatment Lyme disease profiling of antibody, chemokine, and cytokine response pathways is a critical first step to address this complicated question. One of the major limitations for this approach is the lack of appropriate animal models for LNB studies, although a recently developed murine model may offer an accessible option [135]. This mouse model exhibited an increase in the interferon response pathway in the cortex and hippocampus in acute, but not long-term, infection [135]. Interestingly, post-treatment LNB human serum retained elevated interferon levels [175,176]. These mouse and human studies suggest that interferon signaling may play an important role in post-treatment LNB and indeed, could possibly influence the long-term psychiatric symptoms [176]. This begs the question whether additional pathways are also disrupted during long-term LNB infection.

Third, better clinical diagnostic tests for detection of early stages of Lyme disease and better therapeutic treatments for patients affected by PTLDS are needed. Understanding the mechanisms and processes involved with why some patients fully recover and others develop long-term symptoms will be beneficial in addressing these needs. Some recent research suggests that small fiber neuropathy may be associated with PTLDS and might serve as a useful biomarker for evaluating PTLDS in patients [177].

*Borrelia burgdorferi* ss spirochetes regulate Osp expression based on its anatomical location in reservoir hosts and vectors. Therefore, this demands study of LNB in brain tissues, fluids, and cells, as *B. burgdorferi* ss gene expression differs between CSF and blood/skin [74,178,179]. Due to the limitations of accessing tissue from human patients, we propose that LNB is best studied using a mixture of human CSF and post-mortem tissue, in vivo animal infection using murine and non-human primates, and human primary brain cell cultures. There is likely a difference in infectivity of strains and genotypes across hosts, and so any mechanistic studies found in murine or non-human primate models will need to be verified in humans. Nonetheless, we believe that infectious strain specificity can be identified in patient tissues and long-term mechanistic studies can be carried out in laboratory animals. Combined, these works have the potential to influence clinical intervention of the currently untreatable and incurable symptoms of post-treatment LNB. Additionally, prevention of disease is an equally important component to address Lyme disease in public health. By surveilling natural *B. burgdorferi* ss infection in reservoir hosts and tick vectors, we can begin to identify locations with high human health risks of contracting neuroinvasive Lyme disease in North America.

## Figures and Tables

**Table 1:** Currently known human-infecting *Borrelia burgdorferi* strains with corresponding typing classification.

Strain	OspC	RST	IGS	Source
B31, 2-39	A	I	1	Bunikis et al., 2004; Travinsky et al., 2010
2206617	A	I	11	Travinsky et al., 2010
CA4, CA6	A	I	10	Travinsky et al., 2010
BL206, BL203, BL268, B479, B491, B515	A	I	-	Wang et al., 2002
HII, IP1-3, P1F, L5, TXGW, MI2, Pka	A	I	-	Lin et al., 2002
I-24	B	I	3	Bunikis et al., 2004
61BV3, BUR, DK7, Pbre, 35B808, OC2	B	I	-	Lin et al., 2002
64b, B373	Ba	I	3	Travinsky et al., 2010
51405UT	Ba	I	6	Travinsky et al., 2010
ZS7	Bb	I	16	Travinsky et al., 2010
4-55/HB19	I	III	7	Bunikis et al., 2004
OC10	I	I	-	Lin et al., 2002
B500, B331	Ia	III	7	Travinsky et al., 2010
WI91-23	Ia	III	7	Travinsky et al., 2010
CA92-1096	Ib	III	7	Travinsky et al., 2010
297, I-65	K	II	2	Bunikis et al., 2004; Travinsky et al., 2010
149901	K	II	14	Travinsky et al., 2010
272, KIPP, MUL, 28354, OC12-13	K	I	-	Lin et al., 2002

OspC = outer-surface protein C; RST = ribosomal DNA spacer restriction fragment–length polymorphism genotypes (RSTs); IGS = *rrs–rrlA* rDNA intergenic spacer region.
